# Populational Model of *Rhipicephalus microplus* in Beef Cattle in the Southern Region of Paraná, Brazil

**DOI:** 10.3390/vetsci12030206

**Published:** 2025-03-01

**Authors:** Daniel Perotto, Nilceu Lemos da Silva, Meiby Carneiro de Paula Leite, Carolina Fregonesi de Souza, Julio Cesar de Souza

**Affiliations:** 1 Instituto Agronômico do Paraná (IAPAR), Curitiba 80035-270, PR, Brazil; danielperotto@gmail.com (D.P.);; 2 Federal University of Recôncavo Baiano—UFRB, Cruz das Almas 44380-000, BA, Brazil; meiby@ufrb.edu.br; 3 Faculdade de Medicina Veterinária e Zootecnia, Federal University of Uberlândia, FAMEV, UFU, Izaura Augusta Pereira, 286, Uberlândia 38408-192, MG, Brazil; carolina.fregonesis@ufu.br; 4 Federal University of Mato Grosso do Sul, Rua Oscar Trindade de Barros, 740, Aquidauana 79200-00, MS, Brazil

**Keywords:** parasitology, tick, health, animal welfare

## Abstract

In order to describe the population dynamics of *R. microplus* in the southern region of the state of Paraná, Brazil, and to identify racial differences between hosts in the level of infestation by this parasite, the teleogins of approximately 70 males per year were counted every 14 days for 5 years. The herd comprised eight genetic groups, aged 12 to 24 months, in an area of 76 ha (60 ha of native fields and 16 ha of *Hemarthria altíssima*). The monthly averages of the counts per animal were analyzed using the GENMOD procedure, which accounts for the effects of genetic group, month, genetic group × month and cycle. Three generations of ticks of increasing infestation intensity were analyzed in December, February and May, respectively. Animals from Caracu and Canchim breeds as well as Charolais × Caracu and Aberdden Angus × Canchim cross-breeds had the lowest levels of infestation compared to Charolais and Aberdeen Angus. We conclude that by employing the aforementioned mixed breeds, in combination with a strategic control of initiating the application of acaricides in November, followed by two to three treatments every 21 days, this procedure represents the most effective method of controlling ticks in this region.

## Simple Summary

A five-year investigation in Paraná, Brazil, examined tick populations of *R. microplus* on a diverse cattle herd. Tick counts were conducted biweekly to evaluate infestation levels and breed differences. Results indicated that Caracu, Canchim, and their crossbreeds exhibited lower infestations than Charolais and Aberdeen Angus. The study recommends the use of resistant breeds in conjunction with strategic applications. Enhanced host resistance and custom acaricide strategies are essential for effective management.

## 1. Introduction

The genetic improvement of beef cattle in tropical countries has been a longstanding challenge for breeders. The diffusion of European breeds has been presented with obstacles, such as the low adaptability of herds to the local climate and the high incidence of parasites, particularly the *R. microplus* tick. Ticks are Arthropods of the Acari order. In Brazil, there are 61 species of ticks, some of which are of great relevance to both public and veterinary health due to direct illnesses caused during their feeding, and as they can transmit infectious agents to humans and other animals. The *R. microplus* tick, commonly known as the cattle tick, is a hematophagous parasite that consumes between 0.5 and 3 mL of blood in its lifetime [1,2].

Brazil’s vast territorial extension and favorable climatic conditions make it an ideal place for livestock farming, leading to job creation, income generation, and the ability to supply both the national food industry and international markets. In 2018, Brazil was recognized as the world’s largest producer and exporter of beef [2,3].

The country has the second largest cattle herd in the world, with more than 230 million heads. Tick infestations are a challenge for cattle livestock production since they cause significant economic losses, such as a decrease in body weight, milk yield, and diseases, and they are one of the major causes of detriments for cattle producers [4]. Good health and animal welfare conditions are essential to maintain productivity; however, cattle are commonly affected by diseases that compromise their performance [5].

As a result of the complexity of its effects on the host, it is difficult to estimate the economic impact caused by the *R. microplus*. In Brazil, losses associated with this parasite were estimated to be USD 1 billion in 1985. Another study estimated that Brazilian livestock production losses due to tick infestation reached USD 2 billion. Other published data indicate that in Brazil 2.5 million cattle were affected, which represents a loss of 75 million kg of meat, 1.5 billion liters of milk, USD 8.6 billion dollars for secondary damages, and USD 25 million on chemical acaricides to treat tick infestations [1].

In 2014, economic losses caused by the cattle tick were estimated at USD 3.24 billion per year. *R. microplus* also leads to indirect losses due to the cost of chemical control, the accumulation of residue of these products in animal tissues, and the environmental damage it causes [3].

The chemical control of *R. microplus* as an exclusive strategy has proven to be both ineffective and inadequate as it results in the development of tick populations that are genetically resistant to acaricides that are currently available in the market. The emergence and dissemination of resistant tick populations can be accelerated through the large-scale use of chemical products. A detailed review of the mechanisms through which ticks develop resistance to acaricides is available elsewhere. Other problems resulting from the use of chemical acaricides are their environmental impact and their risk to human health, due to the accumulation of their residues in beef and milk [6].

In order to address these issues, a strategic approach is required, focused on an integrated control framework with complementary alternatives to acaricides, such as the use of resistant hosts, natural predators, and changes in their environment. One example of a control strategy to reduce the tick population in pastures is the use of acaricides during the the right time combined with a lower frequency of treatment applications, which is supported by the information about the biology and ecology of *R. microplus* [7].

The southern region of Paraná is situated in a geographically favorable area for the growth and dissemination of cattle ticks; however, systematic studies on this topic are lacking. Thus, this project aims to identify the population model of the tick in this region by quantifying its seasonal prevalence and the degree of infestation in beef cattle of different genetic groups grown in a native pasture.

## 2. Materials and Methods

This study was conducted at the Experimental Station Model Farm of the Agronomic Institute of Parana (IAPAR), located in Ponta Grossa, Brazil. The area occupies 76 ha, 60 of which are native, and the remaining area of cultivated pastures, 8 ha with Coast-cross (*Cynodon dactylon*) and 8 ha with Hemarthria altíssima. The native pastures, which were ploughed between August and September of each year, were accessible to the herds for the entire duration of the experiment, with an approximate occupancy of 0.7 U.A/ha.

The area of cultivated pastures was deferred in mid-February and used for grazing during the critical months of fall and winter. Non-castrated and weaned males of the following breeds were used: Charolais, Caracu, Aberdeen Angus and Canchim, and the reciprocal cross-breedings ♂Charolais × ♀Caracu, ♂Caracu × ♀Charolais, ♂Aberdeen Angus × ♀Canchim and ♂Canchim × ♀Aberdeen Angus. Thus, they comprised a total of 8 genetic groups [8,9].

Approximately 70 bulls per year, aged 10 months and older and tick-free, were used for the experiment: The animals accessed the experimental area during the following five periods: Lot 1 between October 1988 and May 1989; Lot 2 between May 1989 and April 1990; Lot 3 between May 1990 and April 1991; Lot 4 between April 1991 and September 1992; and Lot 5 between September 1992 and October 1993.

During the experimental period, all animals were treated every 14 days with broad-spectrum anthelmintic, in addition to periodic vaccination against aphthous fever and rabies, as well as a polyvalent vaccine against *clostridioides difficile* infection. They also received mineral supplements and a concentrated feed containing 14.5% raw weight and 70% TDN at a dose of 2 kg/animal/day during the whole period, in addition to Coast-cross or corn silage as a source of roughage during winter.

These measures sought to eliminate the interference of other variables, thus allowing the evaluation of the tick’s specific effects exclusively. At the end of each experimental period, the animals to be replaced were kept in a deferred area of cultivated pastures to keep them infested with ticks.

The level of infestation was assessed every 14 days by counting instars between 4.5 mm and 8.0 mm in length, on the same side of the animal’s body. Subsequently, the results of each count were multiplied by 2 to estimate the whole body infestation, and the monthly totals were divided by the number of counts per month to obtain the level of infestation per animal per month.

The data were statistically analyzed using the least squares method through the GENMOD SAS 9.4 (SAS Institute Inc., Cary, NC, USA). The model included the effects of genetic group, the month, and the interaction between genetic group × month and cycle, which was defined as a 12-month period beginning in October and ending in September of the following year. The Gamma distribution was selected, and the logarithmic function was used as the link function.

**Figure 1:**
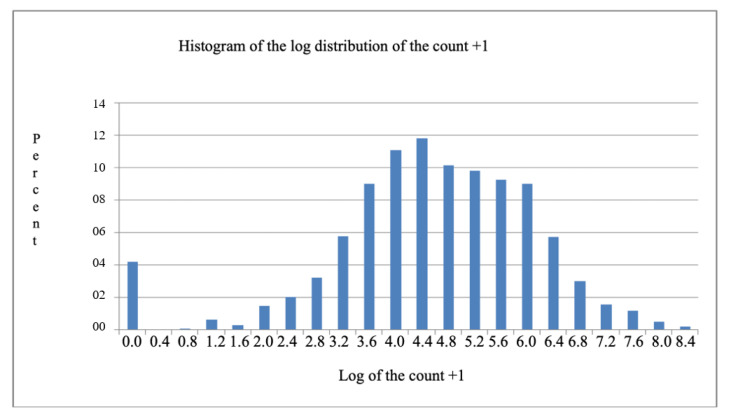
Log distribution of the count + 1 in beef cattle in Campos Gerais, Ponta Grossa, Paraná.

Several authors [10,11] have applied the logarithmic transformation of the count + 1 in order to overcome the limitation of using the analysis of variance when the data in the original scale are not normally distributed. However, in the present case, it is verified that this transformation was insufficient for restoring the normality of the distribution when examining the histogram of the distribution of the log of count + 1 (Figure 1). This occurred because the database contained several observations with the value 0 (zero).

**Figure 2:**
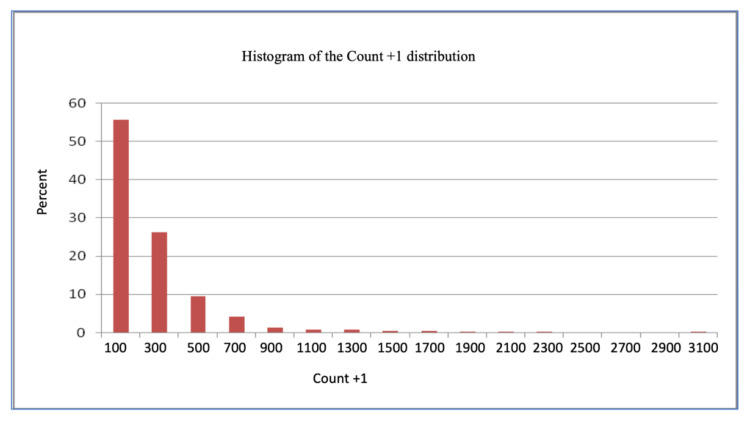
Count + 1 distribution in beef cattle in Campos Gerais, Ponta Grossa, Paraná.

We thus decided to analyze the data in their original scale (count + 1), as shown in Figure 2, by using the Gamma distribution and using the logarithmic function as the link function through the GENMOD method, taking into account the genetic group, the month, and the genetic group × month and cycle (12-month period between October and September). The Gamma distribution was found to be more adequate than the Poisson distribution, possibly due to the large number of observations. This enabled the distribution to be treated as continuous, despite being discreet in its original form.

The analysis included a total of 3640 observations. The results used to assess the suitability of the statistical model are shown in Table 1. The values found for the “Deviance” and “Scaled Deviance” parameters, divided by their respective degrees of freedom, show that the proposed model was suitable to represent the variance of the data. We hope that the answers presented here can be used to guide planning strategies for tick control that will reduce both the costs of treatment and their resistance, in addition to helping to identify breeds and racial compositions of higher bio-economic efficiency for meat production.

## 3. Results and Discussion

The adequacy of the statistical model is presented in Table 1. The values found for the “Deviance” and “Scaled Deviance” parameters, divided by their respective degrees of freedom, show that the proposed model was adequate to represent the variation of the data.

The values presented here can be used to guide planning strategies for tick control that will reduce both treatment costs and tick resistance, in addition to helping to identify breeds and racial compositions with a greater bioeconomic efficiency.

The data were analyzed on their original scale (count + 1), using the Gamma distribution and the logarithmic function as the GENMOD link function in the aforementioned model (Figure 2).

The Gamma distribution proved to be more appropriate than the Poisson distribution, possibly due to the large number of observations. This allowed the distribution to be treated as continuous, despite being discrete in its original form.

All the factors included in the statistical model were of significant importance for the tick counts (Table 2). The differences in the levels of infestation between cycles were marked and varied between 189 teleogins per animal/day during Cycles 1988/89, and 95 teleogins per animal/day during Cycles 1991/92.

The highest levels of infestation were observed during the first 2 cycles, and the lowest in the last 3 cycles. This variation may be explained in part due to differences in climatic factors between one cycle and the next, although the conditions of the pasture and the presence of natural enemies of the tick, such as the cattle egret *(Bubulcus ibis)* and the southern caracara *(Caracara plancus)* may have occurred to reduce the tick population in the final cycles of this study.

Monthly variations in tick populations are influenced by climatic factors, such as temperature, relative humidity, and rainfall, impacting the tick’s free-living stage. Studies have also shown that host infestation levels vary throughout the year. For example, some researchers observed a lower level of infestation during the dry season (April to September) in cross-bred European x Zebu cattle [10,12].

Lemos [11], studying the comparative performance of six Holstein Friesan × Guzera grids in Brazil, produced similar findings in a study assessing the level of tick infestation in animals of six degrees of Holstein × Guzerah blood under field conditions in the same location. Another study using Holstein × Zebu heifers maintained under rotational grazing of elephant grass in Piracicaba, Brazil, also detected a lower parasite load during winter [13].

Spring infestation levels were surpassed by February levels. Starting in spring, the parasite load naturally declined. These results are in agreement with the findings of a study conducted between July 1998 and July 2000 by [10] with Caracu dams originating from two herds, one in the Experimental Animal Production station of the Animal Production in Sertaozinho, and the other from the Caracu Farm in Paranaiba, in the western state of Mato Grosso do Sul.

The first peak of *R. microplus* infestation of engorged females occurred between early September and early October, with an average of 70 ticks on the left side of the animal. A second and more severe peak followed in December, with an average of 168 ticks per animal. Between January and May, infestations were less than 84 ticks per animal. Between June and August, the authors found the highest levels of infestation, with an average peak of 215 ticks per animal in June, and another of 199 ticks per animal in August.

These findings, which contradict most studies on the population dynamics of the *R. microplus*, were attributed to higher-than-normal rainfall in the study year (September 1985 to August 1986). Another study also found higher levels of infestation during the winter months when assessing levels of tick infestation in cross-bred females of European and Indian breeds with predominant Bos taurus blood grazing in *Brachiaria mutica* pastures in Botucatu, Sao Paulo, from September 1995 to August 1998. The authors argued that the hot months were unfavorable for larvae survival, while during the cold months’ conditions improved, resulting in high infestations favored by the higher susceptibility of animals as a result of the lower availability of food due to low temperatures, humidity and rainfall [14].

I work with Jersey × Holstein or Holstein cross-bred dairy cows in the state of Goiás, where the semi-humid climate is characterized by rainy summers and dry winters. Nicaretta [15] found the population dynamics of *R. microplus* from July 2016 to July 2017. Maximum counts recorded in July 2016 (63.18), September 2016 (33.54), November 2016 (17.42), January 2017 (37.46) and March 2017 (43.48).

**Figure 3:**
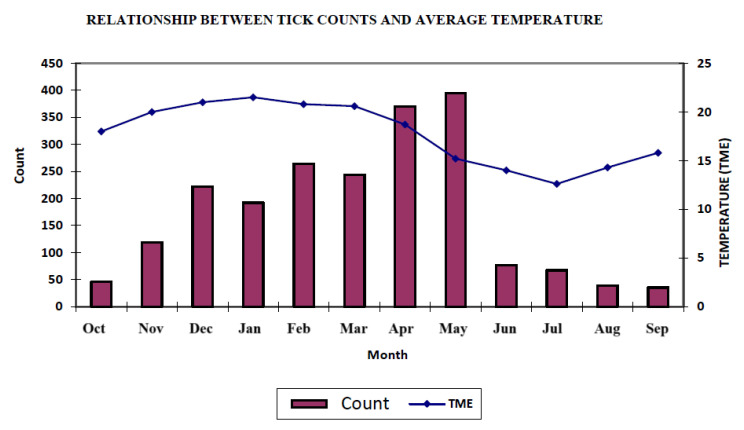
Seasonal variation in tick counts in beef cattle in the southern region of Paraná.

Table 3 presents the average count + 1 by month. Three generations of increasing-intensity ticks were found in December, February, and May, respectively (Figure 3). Three distinct infestation peaks were also reported in a study conducted in Bage, Rio Grande do Sul with Hereford and Ibagé (5/8 Aberdeen Angus + 3/8 Nelore) cattle. The first one was mild and corresponded with the start of infestation in late spring and early summer, followed by a higher intensity infestation in February. Finally, the third peak reflected the highest level of infestation in spring, particularly in April and May [1].

The least square means for the count + 1 according to the genetic group are shown in Table 4. The effects of genetic group on the levels of tick infestation in cattle are widely reported in the Brazilian literature [11].

By working with females of the Red and White Holstein (HVB) and with 1/4, 1/2, 5/8, ¾, and 7/8 HVB × Guzerah cross-breeds in the city of Valencia, Rio de Janeiro, we found that natural tick infestations increased as the HVB fraction of the genetic composition of the cow increased. Under similar conditions and with the same herd, demonstrated that by eliminating 10% of the most highly infected heifers, 18% of the tick population in the HVB group would be eliminated. Eliminating the same proportion of infested animals of the 1/4 HVB × 3/4 Guzerah would eliminate 26% of the tick population [11].

In Argentina, ref. [16] assessed the level of natural infestation by *R. microplus* in the Hereford, Criolla, and Nellore breeds, and in cross-breeds between Hereford × Nellore, finding higher levels of tick infestation in Hereford cattle compared to Nellore, while the Criolla breed was the second highest. The authors concluded that the resistance to *R. microplus* is directly associated with the proportion of Nellore genes in its genetic composition.

The present study shows that the Charolais and Aberdeen Angus breeds are more susceptible to tick infestations than the Caracu breed, while the Canchim (a cross-breed comprising 5/8 Charolais + 3/8 Zebu) had practically half the infestation level of the Charolais, which may be attributed to the higher level of resistance provided by the presence of Zebu in its phenotype. Given that the cross-breed of ♂Charolais × ♀Caracu has a higher proportion of Charolais genes than its reciprocal ♂Caracu × ♀Charolais, as shown in Table 4, this provide evidence that the level of infestation increases proportionately with the fraction of Charolais in the genetic composition of this animal.

Another possibility would be that female Charolais breeds are more susceptible to ticks, infesting themselves with greater amounts of the parasite. The progeny when suckling and touching the mother received a greater load of these (ticks). In the reciprocal crossing (♀Caracu × ♂Charolais), as female Caracu is more resistant to ticks, the calf did not have such a high rate of them. Similarly, ♂Aberdeen Angus × ♀Canchim animals are more susceptible to ticks than the reciprocal cross-breed ♂Canchim × ♀Aberdeen Angus.

**Figure 4:**
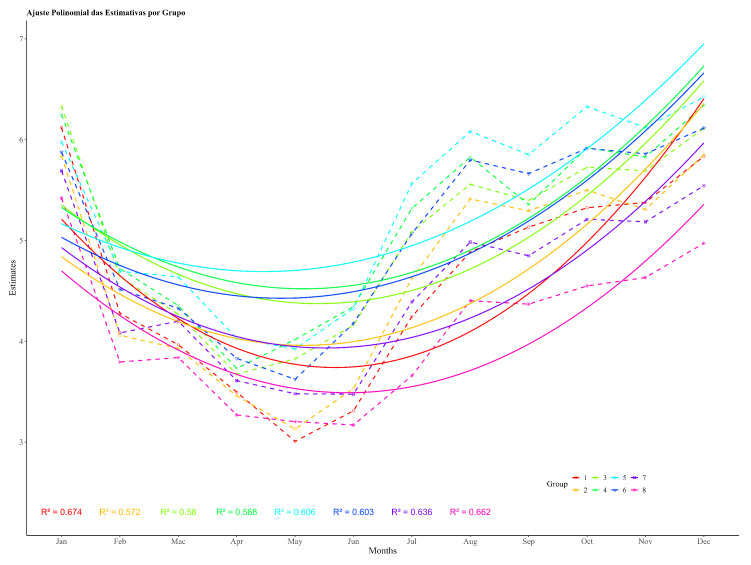
Seasonal variation in tick counts in beef cattle for eight genetic groups: 1. Canchim; 2. Canchin × Aberdeen Angus; 3. Aberdeen Angus × Canchin; 4. Aberdeen Angus; 5. Charlais; 6. Charolais × Caracu; 7. Caracu × Charolais; 8. Caracu in the southern region of Paraná.

Figure 4 shows the population dynamics’ curves of the tick throughout one life cycle for the breeds Aberdeen Angus and Caracu, clearly highlighting the effects of genetic group and month. The differences in the level of infestation between the two groups are small during the months in which climatic factors are unfavorable for larval activity, but large when higher temperatures benefit oviposition, eclosion, and the survival of larvae. The models for population dynamics’ trends also revealed differences between these two breeds, taken as an example of the interaction between genetic groups × month, while the Aberdeen Angus curve is typical of the three generations of ticks described above for the eight genetic groups combined and reported by other studies conducted in the south of Brazil.

The dynamics presented here for the population of the tick in the region, as well as the levels of infestation according to genetic group, suggest the possibility of recommending the integrated control of this parasite through the use of hosts with a higher resistance to it. As reported by Furlong [17], the ideal strategy for controlling ticks in the southern region of Brazil depends on the peculiarity of different sub-regions. However, as shown in Table 3 and by the seasonal variation trend, the first application of acaricides should be in early spring, while the total number of applications, as well as the intervals between them, depend on the results of specific studies with regard to utilizing the optimal control strategy.

Regarding the use of more resistant hosts to the tick, the values in Table 4 show that a significant reduction in infestation levels occurs when the average of the reciprocal cross-breeding is compared with those of their respective European breeds.

For example, the average for the reciprocal cross-breeding of ♂Charolais × ♀Caracu, was 126 teleogins/bull/day, as well as for ♂Caracu × ♀Charolais, while that of the Charolais was 206 teleogins/bull/day. In light of the assessment of the growth traits, food conversion and carcass production of these cross-breeds, in Ponta Grossa, Paraná, beef cattle production based on the use of cross-breeding rotations between Charolais and Caracu results not only in higher production levels than either paternal breed but also in cattle that are more resistant to the tick than the Charolais breed, which may increase the efficacy of chemical treatments. Similarly, studies assessing the productivity characteristics of cross-breeding rotations between Canchim × Aberdeen Angus [8,9] showed that this strategy results in higher meat production in comparison to the use of either paternal breeds.

The average number of teleogins/bull/day of the reciprocal cross-breeds ♂Aberdeen Angus × ♀Canchim and ♂Canchim × ♀Aberdeen Angus (130 ± 1040) was significantly lower than that of Aberdeen Angus (176 ± 1040), once more suggesting that the use of cross-breeds involving breeds of higher resistance to the tick, such as Canchim and Caracu, representing a complementary measure to the use of chemical acaricides in controlling the *R. microplus* tick in cattle.

## 4. Conclusions

Lower levels of tick infestation were found in animals of the Caracu and Canchim breeds. This shows that the choice of cattle breed for different locals is important to reduce tick incidence. Cattle with a tendency to have long hair, such as the Charolais, should be avoided in places with a high incidence of ticks, as they are more susceptible. The use of adapted local breeds such as the Caracu and with a higher degree of Zebu blood, Canchim, showed better performance. The use of these cross-breeds, combined with a strategic control that includes the application of acaricides in November, followed by two or three more treatments every 21 days, represents the most effective method for tick control.

## Figures and Tables

**Table 1:** Criteria for evaluating the suitability of the statistical model.

Criterion	Degree Freedom (df)	Value	Value/df
Deviance	3540	3175.5938	0.8971
Scaled Deviance	3540	4088.2183	1.1549
Pearson $\chi^{2}$	3540	2489.9337	0.7034
Scaled Pearson $\chi^{2}$	3540	3205.5083	0.9055

**Table 2:** Likelihood ratio statistics of Type 3 analysis via the GENMOD procedure.

Source	GL	$\chi^{2}$	Pr > $\chi^{2}$
Month	11	2181.49	<0.0001
Cycle	4	337.18	<0.0001
Genetic group (GG)	7	568.72	<0.0001
GG × Month	77	140.97	<0.0001

**Table 3:** Least square means for tick counting + 1, according to the month of counting in beef cattle, Campos Gerais, and Ponta Grossa-PR.

Month	Cont + 1
October	45 ± 1.057
November	115 ± 1.053
December	216 ± 1.055
January	189 ± 1.055
February	260 ± 1.055
March	245 ± 1.055
April	365 ± 1.050
May	380 ± 1.049
June	77 ± 1.053
July	66 ± 1.054
August	38 ± 1.054
September	34 ± 1.049

**Table 4:** Least squares means for tick counts + 1 in beef cattle in Campos Gerais and in Ponta Grossa-PR, according to the genetic group.

Genetic Group	Count + 1
Canchim	98 ± 1.048
Canchim × Aberdeen Angus	106 ± 1.035
Aberdeen Angus × Canchim	154 ± 1.044
Aberdeen Angus	176 ± 1.040
Charolais	206 ± 1.045
Charolais × Caracu	158 ± 1.045
Caracu × Charolais	95 ± 1.043
Caracu	61 ± 1.042

## Data Availability

The data used in this study cannot be shared because they are part of a research program and are still being used. This may be subject to change in the future.
